# Decision making in *xia*2

**DOI:** 10.1107/S0907444913015308

**Published:** 2013-06-18

**Authors:** Graeme Winter, Carina M. C. Lobley, Stephen M. Prince

**Affiliations:** a Diamond Light Source, Harwell Science and Innovation Campus, Didcot, Oxfordshire OX11 0DE, England; b Faculty of Life Sciences, University of Manchester, 131 Princess Street, Manchester M1 7DN, England

**Keywords:** automation, data reduction, expert system, *xia*2

## Abstract

*xia*2 is an expert system for the automated reduction of macromolecular crystallography (MX) data employing well trusted existing software. The system can process a full MX data set consisting of one or more sequences of images at one or more wavelengths from images to structure-factor amplitudes with no user input. To achieve this many decisions are made, the rationale for which is described here. In addition, it is critical to support the testing of hypotheses and to allow feedback of results from later stages in the analysis to earlier points where decisions were made: the flexible framework employed by *xia*2 to support this feedback is summarized here. While the decision-making protocols described here were developed for *xia*2, they are equally applicable to interactive data reduction.

## Abstract 2

The basis for decision making in the program *xia*2 is described, alongside the framework to support these protocols. Where appropriate, applications of these protocols to interactive data processing are highlighted.

## 1. Introduction   

Careful reduction of diffraction data is key to the success of the diffraction experiment (Dauter, 1999 ▶). This is typically an interactive process, with the crystallographer making use of prior knowledge about the project and data-reduction tools with which they are familiar, as well as a body of knowledge acquired from the analysis of previous data sets. The objectives are to determine the unit cell and symmetry, refine a model of the experimental geometry, measure the reflection intensities and obtain error estimates and correct for experimental effects *via* scaling. In recent years, however, the increase in the throughput of macromolecular crystallography (MX) beamlines has made it difficult to interactively process all data in this way while close to the experiment, giving rise to a need for automated data-analysis software. Several systems have been developed to assist users in these circumstances. Of particular relevance are *Elves* (Holton & Alber, 2004 ▶), *autoPROC* (Vonrhein *et al.*, 2011 ▶) and *xia*2 (Winter, 2010 ▶). More recently, many synchrotron facilities have developed their own MX data-processing ‘pipelines’, for example *XDSME* at Synchrotron SOLEIL, *go.com* at the Swiss Light Source, *fast_dp* at Diamond Light Source, *RAPD* at NE-CAT at the Advanced Photon Source and *AutoProcess* at the Canadian Light Source. Also, the generate_XDS.INP script may be used to run *XDS* with no user input, generating the input on the user’s behalf.

The aim of *Elves* is to allow the user to focus on data collection while the software performs routine data-processing tasks using *MOSFLM* (Leslie & Powell, 2007 ▶), *SCALA* (Evans, 2006 ▶) and tools from the *CCP*4 suite (Winn *et al.*, 2011 ▶). In appropriate circumstances the system is able to proceed from diffraction images to an automatically built structure with essentially no user input, although the *Elves* system includes a ‘conversational user interface’ which allows the user to provide additional information using natural language. The system itself takes the form of a substantial C shell script.

The user interface to *autoPROC* differs from that of *Elves* (and *xia*2) by requiring the user to perform the data-processing task in several steps (Vonrhein *et al.*, 2011 ▶), and it has sophisticated handling of data from Kappa goniometers. *XDSME*, *fast_dp* and *RAPD* are all scripts to automate the usage of *XDS* in a beamline environment and focus on processing of single sweeps of data rather than more complex data sets, which was one of the key requirements of *xia*2.

While many MX data sets consist of a single sequence of images (a single sweep), the general MX data set may consist of more than one wavelength [as necessary to solve the phase problem using the multi-wavelength anomalous diffraction (MAD) technique], each of which may consist of more than one sweep, for example low-dose and high-dose passes, owing to the limited dynamic range of some detectors. The correct treatment in these circumstances is to scale all of the data simultaneously but to merge only those reflections that belong logically to the same set of intensities.

For the processing of diffraction images, the first step is to index the diffraction pattern; that is, to analyse the positions of the observed spots and to determine unit-cell vectors that describe their positions. At this stage, a choice may be made about the Bravais lattice based on the unit-cell parameters and possible space groups may be enumerated. This model of the unit cell and experimental geometry is typically refined before integrating the data, usually employing profile-fitting tech­niques. The subsequent integration step measures the intensities of all of the reflections in the data set. Once all of the data have been integrated in this way, it is necessary to place all of the measurements on a common scale and to correct for systematic experimental effects (scaling) before averaging symmetry-related observations in each wavelength (merging). The scaling requires that the data have the correct symmetry assigned and that they have consistent definitions of the *a*, *b* and *c* unit-cell vectors. In some circumstances the ‘shape’ of the unit cell may be misleading and may suggest a higher symmetry than the true crystal symmetry, and the correct point group may only be determined after the data have been integrated. In this situation it may be necessary to repeat the indexing and integration of the data with the correct symmetry assigned. In the whole crystallographic process the true symmetry may only be known with confidence once the structure has been solved and refined, which is inconvenient given its importance in the initial stages of data processing.

Unlike, for example, *fast_dp*, the *xia*2 system was designed from the outset to support the full complexity possible for an MX data set. In addition, at some stages alternative methods are available; for example, different algorithms and software packages that may be employed (*e.g.* two-dimensional profile-fitting integration in *MOSFLM* *versus* three-dimensional profile-fitting integration in *XDS*). Again, unlike many of the systems described above, *xia*2 embraces the opportunity to offer the user alternative methods with which to process the data. However, in common with much of the software listed earlier, *xia*2 was designed to assist users in performing diffraction experiments as part of the e-HTPX project (Allan *et al.*, 2003 ▶).

A key part of any automated diffraction data reduction will be the implementation of the decision-making processes. In interactive processing these decisions are based on the experience of the user and advice from the program authors, as well as knowledge of the problem in hand. These decisions include the selection of images for the initial characterization of the sample, testing of autoindexing solutions, selection of processing parameters, handling of resolution limits, identification of the point group and selection of a suitable model for scaling. In the development of *xia*2 it was necessary to take a systematic approach to building up this expertise, and a study was undertaken using data from the Joint Centre for Structural Genomics (JCSG; http://www.jcsg.org.) Any results from this are necessarily empirical, as it is impossible to derive the correct choices from first principles. The conclusions of this investigation will be described here, along with the expert system framework in which the decision making was embedded to develop the final data-reduction tool *xia*2.

### 1.1. Data model for macromolecular diffraction data   

The raw diffraction data for MX using the rotation method take the form of sequences of ‘images’ recorded as the sample is rotated in the beam. The minimum useful data set will consist of at least one contiguous sequence of images, hereafter described as a *sweep*, in the majority of cases corresponding to a rotation in excess of 45°. When multiple sweeps are measured the logical structure of the data must be considered: low-dose and high-dose passes logically belong to the same set of intensity measurements. However, when data are recorded at particular wavelengths of a MAD data set each must remain separate, but all must be placed on a common scale to ensure that the *differences* between the observations at different wavelengths are accurately measured. Therefore, the sweeps must be logically grouped into *wavelengths*. Finally, within the *CCP*4 MTZ hierarchy (Winn *et al.*, 2011 ▶) additional groupings of PROJECT and CRYSTAL are defined, with DATASET corresponding to this definition of wavelength. These conventions are also adopted in *xia*2 to ensure consistency with downstream analysis in the *CCP*4 suite.

### 1.2. Workflow of MX data reduction   

**Figure 1:**
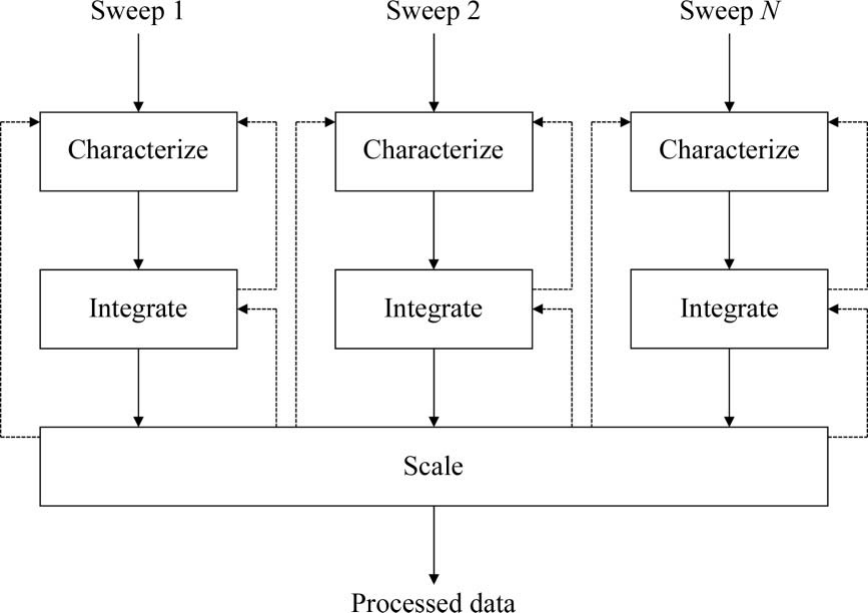
Diagram showing the overall workflow of MX data reduction for multiple sweeps of data, where solid arrows represent the usual path and dotted lines represent feedback to earlier stages. Each must first be characterized before being integrated, after which the data must be scaled, which can also generate feedback.

Taken from the top level, the workflow of data reduction for MX may be considered in three phases: (i) characterization of the diffraction pattern, (ii) integration of data from individual sweeps and (iii) scaling and merging of all sweeps taken from a given crystal. The aim of the characterization of the diffraction pattern is to determine a model for the experimental geometry and a list of possible indexing solutions (described in more detail in §2) and to select a proposal from these. The aim of the integration is to obtain for each reflection the number of ‘counts’ recorded (the intensity), which involves predicting the locations of the reflections and modelling the reflection profiles, after which the intensity is estimated by scaling the model profile to the observed reflection. The precision of this intensity measurement may be estimated from Poisson statistics and the goodness of fit of the model profile. In the final step of scaling and merging these measured intensities are empirically corrected for experimental effects such as variation in the illuminated volume, absorption of the diffracted beams and radiation damage. This flow is summarized in Fig. 1 ▶, in which feedback is shown as dotted lines. Decisions made in the characterization of the diffraction pattern have implications for subsequent analysis, and information from the integration or scaling may contradict these earlier decisions. Any system to automate data reduction must therefore be sufficiently flexible to manage this situation gracefully, considering all ‘decisions’ made about the data set as hypotheses to be subsequently tested, with conclusions only being reached once all tests have been passed. The structure of the system must also mirror this workflow to provide the framework into which to embed decision-making expertise.

The requirement for feedback arises from the relationships between the Bravais lattice, the crystal point group (and incidentally space group) and the unit-cell constants. Strictly speaking, the initial characterization of the diffraction pattern provides a set of triclinic unit-cell vectors which may be used to describe the positions of the observed reflections. The shape of this triclinic basis (*i.e.* the unit-cell constants) may be used to inform the selection of Bravais lattice, typically based on some kind of penalty measuring the deviation from the corresponding lattice constraints (see, for example, Le Page, 1982 ▶; Grosse-Kunstleve *et al.*, 2004 ▶). Selection of a Bravais lattice will have implications for possible point groups as, for example, a crystal with a tetragonal lattice can only have point groups 4/*m* and 4/*mmm*. In the majority of cases a lattice thus assigned will prove to be correct; however, in a small fraction of cases the lattice shape will be misleading; for example, a monoclinic lattice may have β = 90° to within experimental errors. This mis-assignment of the point group may be uncovered in the scaling analysis (Evans, 2006 ▶).

A second cause of complexity may arise when the lattice has higher symmetry than the crystal point group (*e.g.* a tetragonal lattice will have twofold symmetry about the *a* and *b* axes that is not present in point group 4/*m*). In such cases, when more than one sweep of data is included care must be taken to ensure that the sweeps are consistently indexed.

Once the Bravais lattice and point group have been assigned some consideration may be given to the space group. For diffraction data from macromolecular crystals the number of possible space groups for a given point group is typically small and may be further reduced by an analysis of the systematically absent reflections. It is, however, impossible to determine the hand of a screw axis (*e.g.* 4_1_ *versus* 4_3_) from intensity data alone.

### 1.3. Software architecture of *xia*2   

As the workflow of data reduction is expressed in three phases, some of which may be performed with multiple software packages, the framework of the overall system should reflect these phases. Within *xia*2 this is achieved by defining *Indexer*s and *Integrater*s, which act on sweeps of images, and *Scaler*s, which act on all of the data from one sample (*i.e.* all sweeps, organized in wavelengths). Examples of *Indexer*s, *Integrater*s and *Scaler*s may be implemented for every appropriate software package used in the analysis and will embed package-specific information on how to perform the tasks and understand the results. This includes factors such as keyword usage, log-file interpretation and specific decision-making processes. Provided that these definitions are well designed then the different software packages will be functionally interchangeable. The details of this architecture and benefits that it brings are discussed in detail in §[Sec sec5]5.

### 1.4. Blueprint of *xia*2   

The choices made in data reduction may be grouped into two categories: those for which a general selection may prove to be effective and those where the choice must be tailored to the data being processed. The first example could include the specification of the *I*/σ(*I*) threshold for the selection of spots for indexing, where a given threshold tends to work well (Battye *et al.*, 2011 ▶). The second class may be illustrated by the parameters used to define the reflection-profile region, which will be data-set-dependent and where the cost of determining these appropriately is well justified.

The guidelines for the first class of decisions may be determined from an empirical analysis of a large number of data sets recorded in a consistent fashion, which should ideally be free of artefacts such as split spots, sample misalignment during data collection and ice rings, allowing the purely crystallographic decisions to come to the fore. At the time when *xia*2 development began in 2006, the only publicly available substantial source of raw diffraction data meeting these criteria came from the Joint Centre for Structural Genomics, a California-based Protein Structure Initiative consortium, who took the time and effort to make their raw and processed diffraction data available to methods developers. The data sets from the archive used for this study are summarized in Supplementary Table S1^1^ and cover symmetries from *P*1 to *P*622, resolutions from 1.3 to 3.2 Å and unit-cell constants from 30 to 270 Å. These data sets also include MAD, SAD, multi-pass and pseudo-inverse-beam data sets, allowing a wide coverage of likely experiment types. The details of how these were used will be discussed in §§2, 3 and 4.

Finally, it is important to recognize that the software for MX is constantly evolving and that new packages will become available which may be useful to incorporate into *xia*2. As such, some emphasis on abstraction of steps in the workflow is also helpful to allow existing components within the system to be replaced. Currently, *xia*2 includes support for two main integration packages, *MOSFLM* (Leslie & Powell, 2007 ▶) and *XDS* (Kabsch, 2010 ▶), which are accessed as the ‘two-dimensional’ and ‘three-dimensional’ pipelines, respectively, reflecting the approach taken for profile fitting. These are used in combination with *SCALA* (Evans, 2006 ▶), *XSCALE* and more recently (confirming the assertion that new software will become available) *AIMLESS* (Evans & Murshudov, 2013 ▶), as well as other tools from the *CCP*4 suite such as *TRUNCATE* (French & Wilson, 1978 ▶) and *CTRUNCATE*, to deliver the final result. In addition, *LABELIT* (Sauter *et al.*, 2004 ▶) is also supported for autoindexing, offering a wide range of convergence of the initial beam-centre refinement. This was particularly important in the early days of* xia*2 development, when a reliable beam centre in the image headers could not be guaranteed. Finally, the *cctbx* toolbox (Grosse-Kunstleve *et al.*, 2002 ▶) is also extensively used in the analysis to allow lower level calculations to be performed in the *xia*2 code itself.

As the crystallographic data-analysis workflow is considered in three phases within *xia*2, the processes by which the general and specific decision-making protocols were arrived at will be detailed in the next three sections. As the usage of the underlying software packages listed above tends to break down into a two-dimensional and a three-dimensional pipeline (run as xia2 -2d and xia2 -3d, respectively), the specific decision-making protocols for each will be described separately, drawing parallels where possible. Clearly, it is impossible to arrive at rigorously derived protocols as *xia*2 is emulating user decisions. Any justification for the protocols can only be empirical in nature, based on those processes which give the most accurate results for the test data set as a whole.

## 2. Decision making for data reduction: characterization of the diffraction pattern   

Within *xia*2 the *Indexer* carries out the characterization of the diffraction pattern, which consists of an initial peak search and the indexing of the spot list to give a list of possible Bravais lattice options and appropriate unit-cell constants for each, updated values for the model of the experimental geometry and a Bravais lattice/cell proposal, based on an analysis of the possible options. In addition, any crystal orientation matrices calculated should be available. For each program, the choices to make are the selection of images to use for the peak search, the *I*/σ(*I*) threshold to use for indexing, the selection of the ‘best’ solution and analysis to ensure that that selected solution meets the acceptance criteria. It is important to reiterate here that any selection of the best Bravais lattice and unit-cell combination is a proposal to be subsequently tested and does not represent a conclusion drawn.

The primary result of the characterization is a list of Bravais lattice/unit-cell pairs. In cases in which multiple unit-cell permutations are found for a given Bravais lattice (as will be the case for monoclinic lattices for a sample with an approximately orthorhombic cell) it is assumed that the selection with the lowest residual is correct. To date, no counter examples to this assumption have been found. Finally, if subsequent analysis determines that the selected solution is not appropriate, it is the responsibility of the *Indexer* to eliminate this from consideration and provide the next highest symmetry solution for assessment.

### 2.1. *LABELIT* and *MOSFLM*   

*LABELIT* and *MOSFLM* share the same underlying one-dimensional FFT indexing algorithm (Steller *et al.*, 1997 ▶), although the implementation in *LABELIT* allows an additional search to refine the direct beam centre over a radius of ∼4 mm. As such, the behaviour of the programs in indexing is similar, requiring only one analysis for the selection of images with the consideration of solutions dependent on the program used since they have differing penalty schemes. The authors of both *LABELIT* and *MOSFLM* recommend the use of two images for indexing spaced by an ∼90° rotation (Sauter *et al.*, 2004 ▶; Leslie *et al.*, 2002 ▶), giving an orthogonal coverage of reciprocal space from which to determine the basis vectors. A detailed investigation of the effect of the reciprocal-space coverage on the accuracy of the indexing solutions is included in the Supplementary Material. In summary, the use of 1–10 images spaced by 5–90° were scored by means of the metric penalty (Grosse-Kunstleve *et al.*, 2004 ▶) for the correct solution for 86 sweeps from the data sets detailed in Supplementary Table S1, with the conclusion that the use of three images spaced by ∼45° rotation typically gave the most accurate unit-cell constants.

While indexing with *LABELIT* and *MOSFLM* generally shares the same input and output, *MOSFLM* ideally requires an *I*/σ(*I*) threshold for the spot search for indexing. Within *xia*2 this threshold value is obtained by performing an additional analysis of the *I*/σ(*I*) of the spots on the images used for indexing to give at least 200 spots per image to ensure more reliable operation (Powell, 1999 ▶).

In terms of selection of the solution, both *LABELIT* and *MOSFLM* propose an appropriate Bravais lattice and unit cell scored by deviations from the lattice constraints. These suggestions are taken in the first instance, tested and revised as necessary. However, if the user has proposed a unit cell and symmetry from the outset this will override any other decision making.

### 2.2. *XDS*   

Whereas *MOSFLM* and *LABELIT* take spots found on a small number of isolated images, the typical use of *XDS* is to find spots on one or more sequences of images, allowing the centroids in the rotation direction to be calculated (Kabsch, 2010 ▶). Indeed, it is perfectly possible to index with peaks taken from every image in the sweep, a process which may be desirable in some circumstances, and the generate_XDS.INP script developed by the author of *XDS* uses the first half of the sweep by default. Determination of an effective selection of images for indexing with *XDS* is therefore necessary and is described in the Supplementary Material, following a similar protocol to the procedure for *LABELIT* and *MOSFLM*. In summary, the use of reflections from the entire sweep was compared with 1–10 wedges of 1–10° of data spaced by 5–90° by analysis of the metric penalty of the correct solution computed from the triclinic cell constants from indexing using tools from *cctbx*, with the conclusion that the use of three wedges of data each of ∼5° and spaced in excess of ∼20–30° was found to give the most accurate results. The authors of this manuscript note that this conclusion is based on essentially good quality data from a structural genomics programme and that this may not be appropriate for weaker data. In this case, the user may instruct the spot search to be performed on every image in the sweep by providing the -3dii command-line option to *xia*2.

When characterization is performed with *XDS* it is necessary to decide which of the 44 ‘lattice characters’ may correspond to a reasonable solution. In the default use, as expressed by generate_XDS.INP, no selection is made and all integration is performed with a triclinic basis. The *XDS* indexing step *IDXREF* outputs a penalty for each of the 44 lattice characters, normalized to the range 0–999, and in this study no examples were found where the correct solution had a penalty of greater than 40 given an accurate beam centre. Therefore, when no additional guidance is provided to *xia*2 all solutions with a penalty lower than this will be considered, removing duplicate lattice solutions, and the highest symmetry solution will be proposed for subsequent analysis.

### 2.3. Summary   

The protocols for the selection of images for autoindexing were found to have a high degree of commonality given the disparate algorithms used. All autoindexing schemes seek out an appropriate set of unit-cell vectors which describe the reciprocal-space coordinates of the peaks observed on the images. If a single image or narrow wedge of images is used, at least one direction will be represented only as linear combinations of basis vectors. However, if three well spaced images or wedges are used the coverage of reciprocal space is more complete, increasing the likelihood of observing the basis vectors in isolation and hence more accurately reporting their directions and lengths.

While *XDS*, *MOSFLM* and *LABELIT* are all supported for the initial characterization stage in *xia*2, *LABELIT* is chosen by default (when available) as the beam-centre refinement was found to make the analysis process as a whole more reliable. While the output of *LABELIT* is entirely compatible with *MOSFLM* and can be used immediately (Sauter *et al.*, 2004 ▶), the assumptions made about the experimental geometry are inconsistent with the more general model implemented in *XDS* (Kabsch, 2010 ▶). In the situation where *XDS* is to be used for integration while *LABELIT* has been used for characterization, the indexing will be repeated with *XDS*, taking the refined model from *LABELIT* as input.

## 3. Decision making for data reduction: integration   

**Figure 2:**
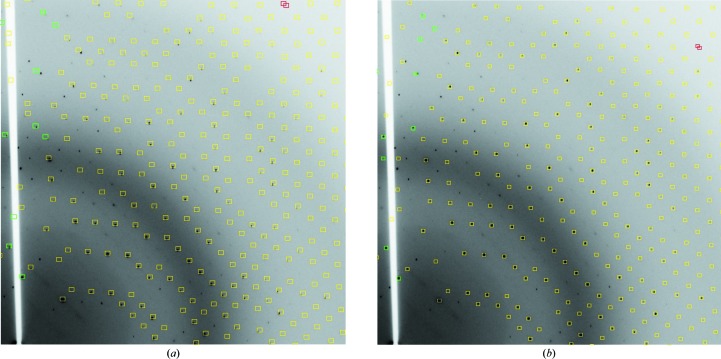
One quadrant of a diffraction image recorded for JCSG sample 12847 (PDB entry 1vr9) which has close to *I*222 symmetry. The image in (*a*) has been indexed and refined with *iMosflm* imposing orthorhombic lattice constraints, while the image in (*b*) has been indexed and refined imposing monoclinic constraints. While the pattern in (*b*) is clearly better predicted, both were ‘successfully’ processed in subsequent interactive integration, although the intensities integrated with a monoclinic lattice were much more accurately measured.

The objective of the integration step is to accurately measure the intensities of the reflections. During this process the results of the characterization will be tested and a refined model of the experimental geometry and crystal lattice will be determined. While the first objective of accurately measuring the reflection intensities must be the focus of the integration, for an expert system the validation of the initial characterization is also significant, since images are not inspected to ‘see’ whether something is wrong, unlike a user performing interactive processing. This is illustrated in Fig. 2 ▶, where data from JCSG sample 12487 (PDB entry 1vr9) with *C*2 symmetry close to *I*222 have been indexed and refined with *iMosflm*. From simply inspecting the alignment of the measurement boxes with the spots on the image characterized with an ortho­rhombic body-centred (oI) lattice (Fig. 2*a*) it is clear that the pattern is not well modelled, particularly when compared with the same image characterized with a monoclinic centred (mC) lattice (Fig. 2*b*).

In addition to the testing of the characterization, the system must emulate the behaviour of an expert user, perhaps working with a graphical user interface. This will lead to a certain amount of additional bookkeeping that may otherwise be handled by the graphical interface, and will be discussed below. For *XDS* this is less of an issue, as the command-line interface is used exclusively by the interactive user as well as *xia*2, although images are output by *XDS* for viewing with *XDS-Viewer* for diagnostic purposes.

Finally, while the results of *XDS* characterization are sufficient to allow integration to proceed, with *MOSFLM* some additional preparation is necessary, in particular refinement of the unit cell and experimental geometry. As such, the integration process is explicitly separated into preparation for integration and integration proper, with the cell refinement playing a major part in the preparation phase.

### 3.1. Two-dimensional pipeline: integration with *MOSFLM*   

A typical interactive integration session with *iMosflm* starts with indexing followed by refinement of the cell. By working through this process a significant amount of information about the images is accumulated by *iMosflm*; for example, spot-profile parameters from the peak search prior to indexing. In automating this analysis, particularly when alternative programs may be used for some of the steps, a specific effort must be made to reproduce this information. This process, coupled with the cell-refinement step, is characterized in the *xia*2 two-dimensional pipeline as ‘preparation for integration’.

#### 3.1.1. Preparation for integration with *MOSFLM*   

The task of preparing for integration is threefold: to refine the unit-cell constants and experimental geometry, to emulate the process the user performs using *MOSFLM* *via* *iMosflm* and to perform some analysis of the results of characterization. Preparation includes using a subset of the available data for cell refinement. Consequently, the optimal subset of data needs to be selected. Within *xia*2 this choice of subset is made in a manner identical to the selection of images for *XDS* characterization, with the additional constraint of using at most 30 frames (a limitation in *MOSFLM* when this decision was studied and implemented). In essence, the cell refinement is performed without lattice constraints (*i.e.* in *P*1) using 1–10 wedges of three images uniformly distributed in the first 90° of data followed by a test using 2–10 images per wedge and a spacing of 10–45°. The conclusion was that the use of three wedges of three images, with spacing as close as possible to 45°, gave the most accurate cell constants. The full results of this analysis are included in the Supplementary Material. It is important to note that this procedure emphasizes the stability of refinement in the absence of lattice constraints.

The cell-refinement step also emphasizes some of the more procedural requirements of integration with *MOSFLM*. In particular, when *MOSFLM* is used for autoindexing the spot search determines reasonable starting parameters for the reflection-profile description. If *MOSFLM* is not used, it will be necessary to obtain these initial profile parameters elsewhere. Within *xia*2 this is achieved by performing a peak search on the first frame of each wedge used for cell refinement. The peak search also provides a conservative estimate of the resolution limit, which is applied during the cell-refinement step.

To replace the visual analysis of the characterization, as highlighted in Fig. 2 ▶, an algorithmic approach is needed. Initially, the cell refinement was performed without lattice constraints, and the refined cell parameters with their error estimates reported by *MOSFLM* were compared with the lattice constraints. The estimated standard deviations were found to be rather unreliable (for example, refining β to 7σ away from 90° for a tetragonal lattice) and the approach does not emulate a visual inspection of the images. Equally, analysis of the positional deviations between the observed and the predicted spot positions was also unreliable, as sample quality makes a significant contribution to, for example, the spot size.

The approach developed for *xia*2 does in fact use the root-mean-square (r.m.s.) deviations between the observed and predicted spot positions, but ‘normalizes’ this by performing the cell refinement with and without the lattice constraints and comparing the deviations in a pairwise manner as a function of image and refinement-cycle number. If the lattice constraints are appropriate the deviations should be at least as good in the constrained case as in the unconstrained case. If the r.m.s. deviations are made substantially worse by applying the Bravais lattice constraints it is unlikely that the lattice constraints are appropriate. The ratio of the r.m.s. deviations with and without the lattice constraints was lower than 1.5 in all cases where the correct solution was chosen. In the example shown in Fig. 2 ▶ this ratio was 2.06 for oI and 1.04 for mC, showing that the method is effective at least for this example. It is useful to note that the data may be processed (poorly) in *I*222 with *iMosflm* (including the *POINTLESS* analysis and the quick scaling with *AIMLESS*) simply by pressing ‘ignore’ once: *POINTLESS* does not identify the incorrect point-group assignment. For reference, the correct β angle for the lattice in the mI setting is 90.6°.

#### 3.1.2. Integration with *MOSFLM*   

Once the preparation for integration is complete, the integration itself may proceed. There are a number of choices to be made in terms of how to perform integration, as well as some procedural considerations. The choices to make are whether to perform the integration applying the lattice constraints (as recommended by the authors of *MOSFLM*; Leslie & Powell, 2007 ▶), whether to refine cell parameters during integration (not usually recommended by the authors of *MOSFLM*) and whether to re­integrate the data with the final resolution limit applied as opposed to integrating all reflections across the detector face.

The procedural aspects of performing integration are straightforward. Some careful bookkeeping is needed to ensure that the program state at the end of successful cell refinement is reproduced at the start of integration (to be specific: the sample misorientation angles, experimental geometry and reflection-profile parameters.) In addition, if parallel integration is desired some additional refinement of the sample-misorientation angles at the start of each sweep will be needed to ensure that no discontinuities are introduced into the integration model between processing blocks.

To address the specific, rather than procedural, choices for integration of the data, 38 sweeps from the 86 used in the characterization study (corresponding to 12 JCSG data sets) were integrated in their entirety several times. The assessment of the quality of the resulting intensities was performed as follows: the data from each integration run were reindexed and sorted in the correct point group with *POINTLESS* and the data were scaled with *SCALA* (as *AIMLESS* did not exist at the time) using smoothed scaling on the rotation axis with 5° intervals, secondary-beam correction (with six orders of spherical harmonics) and smoothed *B*-factor correction with 20° intervals (the default in *CCP*4*i*). These corrections allow the overall intensity of the diffraction to be corrected over relatively short intervals, allow empirical absorption corrections and allow slow decay owing to radiation damage, all of which are appropriate for the data sets used in the study. The overall *R*_merge_ for the data set was used as the test metric; this was justified as follows. Firstly, the resolution limit and the extent of the data set were unchanging, so the multiplicity-dependence of the statistic was not relevant. Secondly, the *R*_merge_ is directly related to the *I*/σ(*I*) of the data (Weiss & Hilgenfeld, 1997 ▶) when the σ(*I*) estimates are reliable. Here, however, the σ(*I*) estimates may not be reliable, but the *R*_merge_ calculation itself has no σ(*I*) dependence, making it a more reliable indicator than *I*/σ(*I*). Also, it may be expected that better choices for the integration would result in improvements in the measured intensities, which should in turn result in better agreement with symmetry-related observations after scaling.

The protocols tested, as summarized in Table 1 ▶, were (1) the procedures recommended by the authors of *MOSFLM*, integration allowing refinement of the unit cell with (5) and without (2) the application of the lattice constraints, integration with a triclinic lattice but with fixed unit-cell constants (3) and the recommended procedure but with the final resolution limit additionally imposed (4). The results of these runs are also summarized in Table 1 ▶, which shows the average *R*_merge_ normalized to the first integration procedure and the number of successful and failed runs. Reassuringly, the recommended procedure gives results as good as any other, with those protocols not recommended by the authors of *MOSFLM* proving to be unreliable and giving poor results when successful. The conclusion from this is to fix the cell constants, apply the lattice constraints and to integrate reflections from the entire active area of the detector, applying the resolution limit later in scaling. Two final procedural choices are made: if *MOSFLM* provides an updated value for the detector gain in the warnings at the end of the output this updated value is used and the integration is repeated. Also, if the integration fails owing to an error in the refinement of the measurement-box parameters it is repeated with these parameters fixed at their values from the end of the cell refinement.

### 3.2. Three-dimensional pipeline: integration with *XDS*   

Whereas *MOSFLM* is typically run through the graphical user interface *iMosflm*, *XDS* is run on the command line with instructions in a text file. In addition, almost all of the intermediate files are written as plain text and are well described in the program documentation, making *XDS* very well suited for implementation within an automated system. The authors of *XDS* also provide generate_XDS.INP, which can be used to compose an *XDS* input file from the headers of a sequence of images. This will instruct *XDS* to autoindex from peaks found in the first half of the sweep, perform all integration with a triclinic basis and decide the appropriate Bravais lattice and point group at the scaling stage. By default, no parameter recycling is performed, although the methods for parameter recycling are well documented. At the time when this study was performed, however, generate_XDS.INP did not exist.

The aim here is to determine the processing choices which will generally give good results with *XDS* for use within *xia*2. Ideally, these should not impose an excessive computational cost, for example more than doubling the processing time. As integration with *XDS* is already largely automatic, only a small number of choices need to be made; namely, whether to impose the Bravais constraints during processing and to what extent to ‘recycle’ experimental parameters after processing. The latter may include the reflection-profile parameters, the globally refined orientation matrix and experimental geometry and the local detector distortions from post-refinement (which may be assumed to be ‘small’ for image-plate, corrected CCD and pixel-array detector images). As with the analysis for *MOSFLM*, *R*_merge_ will be used to assess the benefit or otherwise of these choices compared with the straightforward process as embodied in the generate_XDS.INP instructions.

The results of integrating the 38 sweeps using the five integration protocols are summarized in Table 2 ▶, from which two results are clear: all of the protocols make insignificant differences in the accuracy of the measured intensities (for good data) and the processing is generally reliable in all cases. The only choice that had a measurable benefit was the recycling of the reflection-profile parameters that define the transformation of the data from the image to reciprocal space. To apply these refined values requires that the integration of the data set is repeated. As the recycling of the GXPARM file made little difference but caused no problems, this is also used in the repeated integration. The same approach was taken for the Bravais lattice constraints, as they may improve the results for poorer quality data.

With the integration with *MOSFLM*, post-refinement was used to assess the appropriateness of the Bravais lattice constraints. With *XDS* the post-refinement is performed after the integration is complete; however, the approach of challenging the lattice assignment *via* post-refinement remains valid. Whereas *MOSFLM* provides r.m.s. deviations of the spot positions as a function of every frame and cycle, *XDS* provides only an overall r.m.s. deviation in position and rotation at the end of refinement. Therefore, for the *XDS* output these r.m.s. deviations are divided by their unrestrained counterparts and added in quadrature to give the overall score. As with *MOSFLM* no correctly assigned lattices were found with this ratio exceeding 1.5, and the pseudosymmetric lattice showed in Fig. 2 ▶ had a ratio of 2.6. Therefore, 1.5 is taken as the limit on acceptable values, and no counterexamples have been found to date.

### 3.3. Summary   

The decision-making protocols for the integration of diffraction data with *MOSFLM* and *XDS* have been presented. For *MOSFLM* this is split into two phases, essentially the cell-refinement step and integration, with the former used to test the assignment of the Bravais lattice. A key finding was that performing the cell refinement with three wedges of images spaced by ∼45° was found to give the most reliable refinement. For integration itself the conclusion was that the protocol recommended by the authors of *MOSFLM* of applying the lattice constraints, fixing the unit-cell constants and integrating across the entire detector face was suitable. For integration with *XDS* small improvements were found from recycling some of the processing parameters; however, all protocols proved reliable with good quality data. This may explain the popularity of *XDS* within the beamline automated data-processing systems listed earlier. Finally, while tools such as generate_XDS.INP can be used to run *XDS* for integration in an automated manner, they are limited in terms of scaling more than one sweep

## 4. Decision making for data reduction: scaling   

The objective of *xia*2 has always been to process a data set consisting of one or more sweeps from diffraction images to scaled and merged intensities. When the entire data set consists of one sweep, this is relatively straightforward. When the data set consists of more than one sweep, substantially more work is needed (as illustrated in Fig. 1 ▶) as the integrated data must be tested for consistency in the Bravais lattice choice and indexing basis. The point group determined for the measured intensities for each sweep must also be consistent with the Bravais lattice choice used for processing. These procedures require careful management of derived data and must allow the possibility of feedback to earlier stages in the data analysis. To help with this, the scaling process is split into three phases: preparation for scaling, the scaling itself and the subsequent analysis.

Given the use of *MOSFLM* and *XDS* for integration, *SCALA*/*AIMLESS* and *XSCALE* are used for scaling. In addition, substantial use of *POINTLESS* is made within the preparation phases and of (*C*)*TRUNCATE*, *CAD* and other tools within the *CCP*4 suite for subsequent analysis. For scaling data from *XDS* additional procedural decisions are needed, namely whether to scale the data in both the *XDS* CORRECT step and *XSCALE* and how to merge the scaled data.

### 4.1. Preparation of data for scaling   

**Figure 3:**
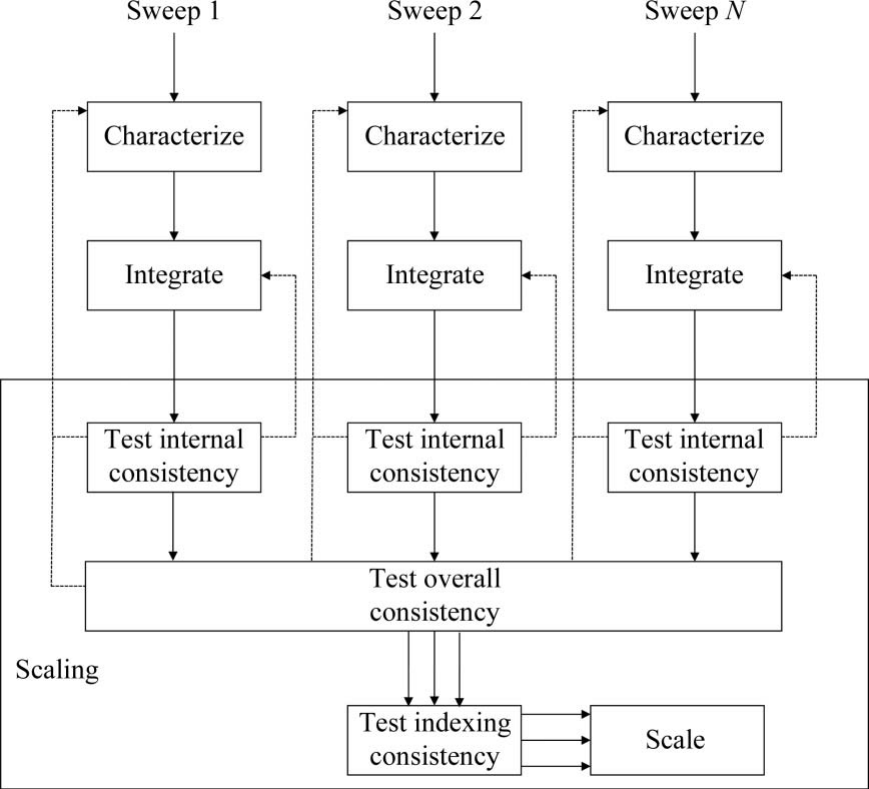
Overall workflow for the preparation for scaling showing details of how the scaling is performed, where solid lines represent the flow of data for each sweep and dashed lines represent feedback. The details of the tests are shown in Figs. 4 ▶ and 5 ▶.

**Figure 4:**
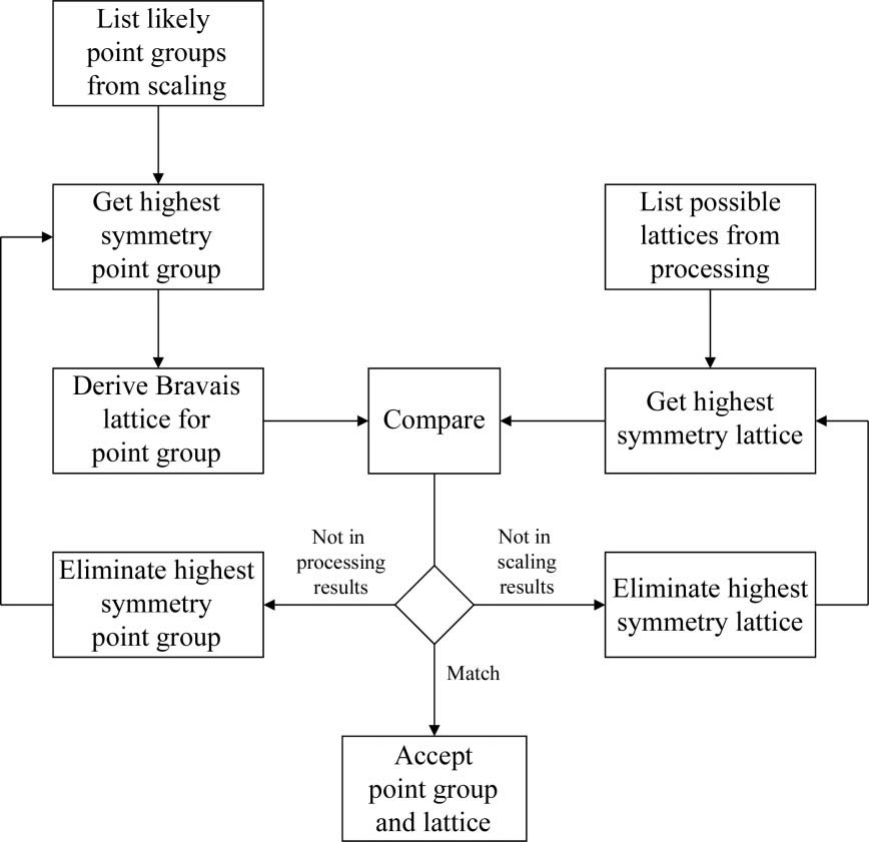
Procedure to determine the crystal point group and Bravais lattice consistent with the diffraction data, taking into account the results from indexing and analysis with *POINTLESS*. Decisions are shown as diamonds and processing tasks as rectangles

**Figure 5:**
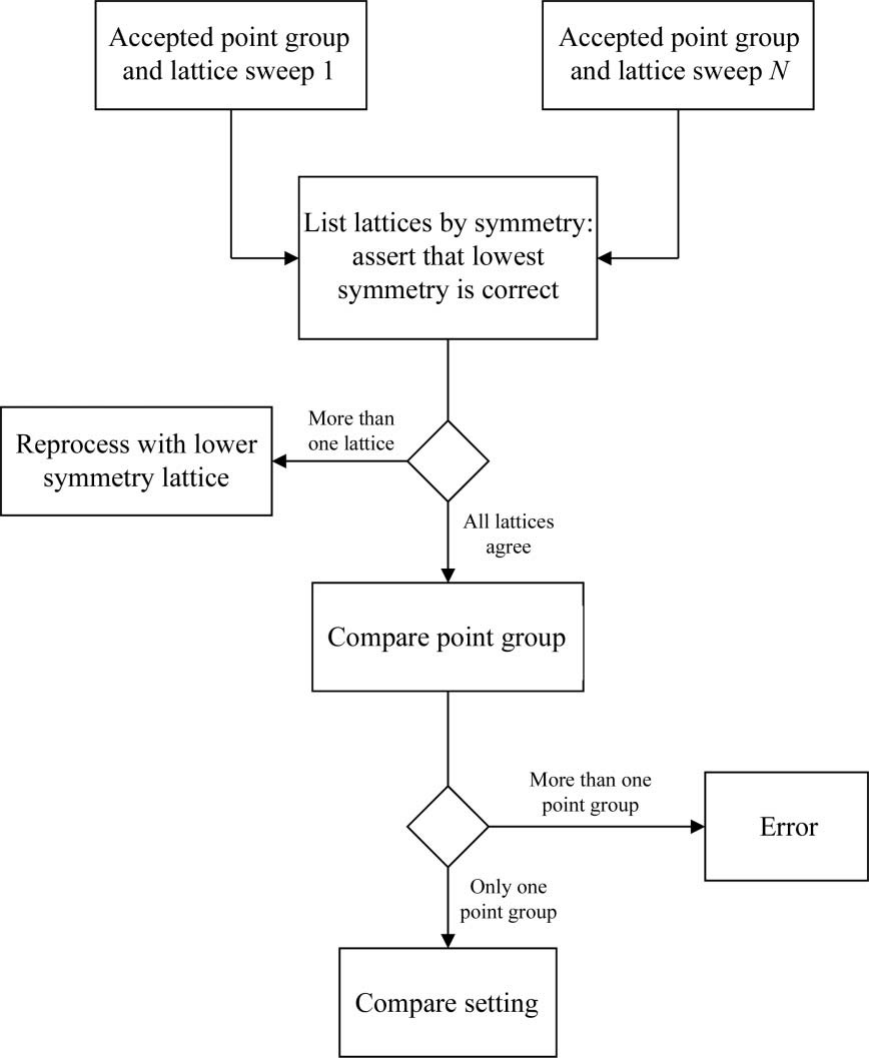
Procedure for combining point-group information from all sweeps, following on from Fig. 4 ▶, making the assumption that the lowest symmetry lattice is correct.

The purpose of the preparation phase in scaling is to test the measured intensities for consistency with the Bravais lattice within and between each sweep of data and to ensure that all related sweeps have the same point-group assignment and indexing convention. The overall ‘flow’ of the preparation is shown in Fig. 3 ▶ and has embedded procedures for testing the internal consistency (Fig. 4 ▶) and the consistency between sweeps (Fig. 5 ▶.) The first test determines whether the Bravais lattice used for processing is consistent with the point-group symmetry in the measured intensities using *POINTLESS*. If the selection is consistent then the test is passed. If the lattice corresponding to the symmetry of the data has a lower symmetry than the lattice used for integration, integration will be repeated with the lower symmetry lattice. If the proposal for the point group has higher symmetry than the lattice used for integration it is ignored and the next proposal is considered. The latter is necessary to cope with pseudosymmetric data, where the higher symmetry possibility has already been excluded. For example, the test for internal consistency will identify *P*2 data with β = 90.0°.

The purpose of the test for overall consistency is to ensure that the data have been processed with uniform unit-cell axis definitions. In many cases this will not be in question, but in some circumstances (for example, pseudosymmetric data sets with low-resolution and high-resolution passes) it may be necessary to propagate information from one sweep to another. Finally, once all of the data have been processed with a consistent lattice and have consistent point-group assignments it is necessary to ensure that a consistent indexing basis is used. In situations in which the Bravais lattice has higher symmetry than the point group it is possible to assign equally valid but inconsistent basis vectors. For scaling it is critical that these are consistently defined so that *xia*2 compares the indexing of second and subsequent sweeps with the first using *POINTLESS* and the data are reindexed as required. These preparation for scaling steps are common to both the two-dimensional and the three-dimensional pipelines.

### 4.2. Two-dimensional pipeline: scaling with *SCALA*   

The approach to scaling performed by *SCALA* and *AIMLESS* is to determine a parameterized empirical model for the experimental contributions to the measured intensities and then to adjust the parameters of this model to minimize the differences between symmetry-related intensity observations. The recommended protocol for scaling (as expressed in *CCP*4*i*; Evans, 2006 ▶) is to have the overall scale smoothed over 5° intervals, to allow an isotropic *B*-factor correction smoothed over 20° intervals (for radiation damage) and to have an absorption surface for the diffracted beam parameterized with six orders of spherical harmonics. While this model works well, there are examples where including additional corrections (*e.g.* the TAILS correction for partial bias) may substantially improve the level of agreement between the observations. As such, a decision is needed as to the most appropriate scaling model to apply.

**Figure 6:**
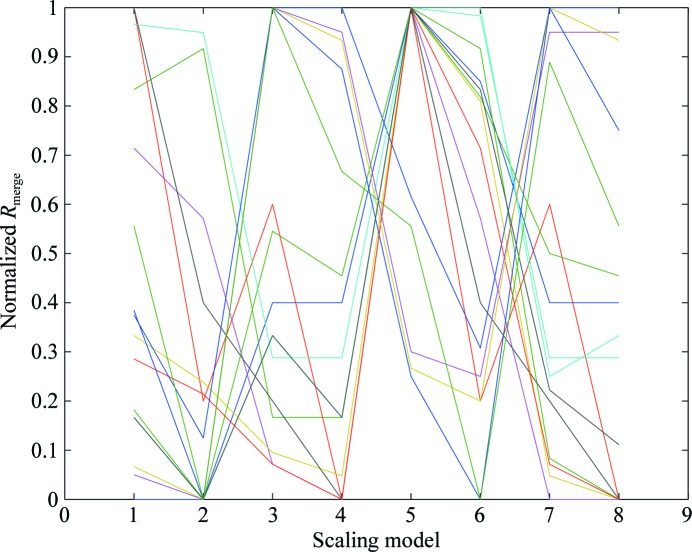
*R*_merge_ values for each of the 12 JCSG data sets used (with a different colour for each) for all eight permutations of the scaling model shown in Table 3 ▶, normalized to the range 0–1 (lowest to highest). Clearly, no single model systematically gives the lowest residual and almost all models work well for at least one example and are hence worth considering.

Initially, an investigation was performed for 12 JCSG data sets, testing eight scaling models with each, as summarized in Table 3 ▶. The *R*_merge_ values for each were normalized to the range 0–1, where 0 corresponds to the lowest and 1 to the highest. As is clear from Fig. 6 ▶, no single model reliably gave the lowest merging residuals. The conclusion was therefore that the optimum scaling model must be determined for each case. Within *xia*2 this process is implemented by initially allowing five cycles of scale refinement and is scored by *R*_merge_ in the low-resolution shell and the convergence rate. The low-resolution *R*_merge_ is used as the scale factors for the data set are dominated by the strong low-resolution data and the extent of the data set is unchanging (*i.e.* the usual criticism of *R*_merge_ does not apply). In addition, the low-resolution data contribute to all elements of the scaling corrections owing to the way in which the corrections are parameterized, making the process robust. Once the scaling model has been selected more cycles are allowed for full scaling.

Resolution limits are calculated following the procedures set out in §[Sec sec4.4]4.4 based on an analysis of the intensities after the initial scaling. In terms of the fine-grained parameterization (*e.g.* the rotation spacing for the *B*-factor correction) little benefit was found in deviating from the defaults. Finally, early versions of *xia*2 included an iterative remerging protocol to refine the error-correction parameters to obtain χ^2^ = 1. As both *AIMLESS* and *SCALA* now perform this refinement this now-redundant process was removed.

### 4.3. Three-dimensional pipeline: scaling with *XSCALE*   

*SCALA* and *AIMLESS* use a parameterized model to determine the scale factors for each reflection. In contrast, *XDS* and *XSCALE* use arrays of correction factors to remove the correlation of the measured intensities with image number and detector position (Kabsch, 2010 ▶). Correction factors are applied for sample decay, absorption and detector sensitivity, and the combination of corrections to apply is under the control of the user, although the default if no instruction is given is to apply all corrections.

In the *XDS* CORRECT step the corrections are determined for each sweep in isolation, whereas the corrections are jointly refined for all sweeps in *XSCALE*. When this was investigated during *xia*2 development little benefit was observed in scaling the data twice (*i.e.* in the *XDS* CORRECT step and in *XSCALE*) as this doubled the number of correction factors, so the choice was made to scale the data only in *XSCALE*. Subsequent discussion with the authors of *XDS* highlights that this apparent doubling of the number of correction factors may be misleading (Kay Diederichs, private communication).

In terms of the choice of corrections to apply, it was found that, unlike in *SCALA* and *AIMLESS*, applying all of the possible corrections always gives the lowest residual. Consequently, all corrections are applied in *xia*2, with a user option to override this decision. After initial scaling the resolution limits are determined following the procedures set out in §4.4, after which the scaling is repeated with the limits applied. The scaled intensities are then output unmerged, converted to MTZ format and merged with *SCALA* or *AIMLESS* to generate a report of merging statistics.

### 4.4. Resolution-limit calculation   

One of the key decisions to make in MX data reduction is the assignment of a resolution limit. Too low a limit will result in throwing away useful data, while too high a limit may not improve the structure solution and refinement and may suggest excessive precision in the resulting atomic coordinates. Historically, a wide range of heuristic criteria have been used to decide the high-resolution limit of the data, including thresholds on the *I*/σ(*I*), *R*_merge_ and completeness. Recent systematic studies suggest that methods based on correlation coefficients may give a more robust insight (Evans & Murshudov, 2013 ▶; Karplus & Diederichs, 2012 ▶); however, these have yet to be implemented in *xia*2.

Within *xia*2, the *I*/σ(*I*), *R*_merge_ and completeness may be used as criteria for resolution-limit determination. By default the merged and unmerged *I*/σ(*I*) are used, with thresholds of 2 and 1, respectively. While the former reflects a ‘digest’ of several CCP4 Bulletin Board discussions on the subject of resolution limits, it is important to note that the user has complete control over the resolution-limit criteria. The latter limitation becomes more significant with high-multiplicity (tenfold or more) data. To be specific: during the development of *xia*2 it was found that in high-multiplicity cases an *I*/σ(*I*) of 2 may correspond to an unmerged *I*/σ(*I*) of <0.5. In these cases it was found that the merged intensity measurements in the outer resolution shells tended towards a normal distribution rather than the expected exponential distribution (as assessed by the statistic *E* ^4^), suggesting that the measurements were dominated by noise. While this result may indicate a poor treatment of the experimental errors for weak reflections, the choice was made to additionally impose a limit on the unmerged *I*/σ(*I*) to ensure that the reduced data were reliable. This has the side effect of typically limiting the *R*_merge_ in the outer shell to be less than 100%. The authors note that a treatment of resolution limits based on correlation coefficients would not suffer from these issues.

To calculate the resolution limits, early versions of *xia*2 used an analysis of the *SCALA* log-file output. This was found to be sensitive to the choice of resolution bins, so more recent versions of *xia*2 compute the merged and unmerged *I*/σ(*I*), the *R*_merge_ and the completeness directly from the scaled but unmerged reflection data and fit an appropriately smoothed curve before identifying the limit. In addition to having fine-grained control over the resolution-limit criteria, the user may also set an explicit resolution limit. Finally, inclusion of the option of correlation coefficient based resolution limits is planned for the near future.

### 4.5. Post-processing   

Although the primary objective of *xia*2 is to arrive at correctly integrated, scaled and merged intensities, there are a small number of downstream analysis steps which make the system more useful to the user by providing data files ready for immediate structure solution and refinement. The analysis steps are to calculate structure-factor amplitudes from the intensities following the *TRUNCATE* procedure (French & Wilson, 1978 ▶) as implemented in *CTRUNCATE*, to perform local scaling using *SCALEIT* (Howell & Smith, 1992 ▶) for data with more than one logical wavelength and to determine an ‘average’ unit cell for downstream analysis. Additionally, reflection-file manipulation is performed with *CAD* from the *CCP*4 suite and the data are assessed for twinning using methods included in *cctbx*.

The calculation of structure-factor amplitudes from intensities fundamentally consists of a scaled square root. The scale factor reflects the fact that the intensities are recorded on an arbitrary scale while the structure-factor amplitude scale depends on the contents of the unit cell (Wilson, 1942 ▶). The square root corresponds to the fact that the intensities are proportional to the square of the structure-factor amplitude. The *TRUNCATE* procedure is a treatment for measured negative intensities, computing the most likely value for the true intensity given the positivity constraint. For this to be correctly applied it is critical that the systematically absent reflections are removed. Absent reflections resulting from centring operations (*e.g.* *h* + *k* + *l* odd for body-centred systems) are excluded during integration based on the Bravais lattice choice. To remove those resulting from the screw axes of the space group it is first necessary to identify a space group consistent with these absences: for this *POINTLESS* is used. While the result of this analysis is typically not unique, it is assumed that the result should be appropriate for reducing the bias in the *TRUNCATE* procedure. The user should be aware that although the point-group assignment from *xia*2 is typically reliable, the space-group assignment may be incorrect and is also reliant on the axial reflections having been recorded. A list of all likely space groups (*i.e.* those consistent with the observed systematic absences) is provided in the console output.

If a sequence file is found alongside the diffraction data or is provided in the input file, an estimate of the number of molecules in the asymmetric unit is made following a probabilistic procedure (Kantardjieff & Rupp, 2003 ▶) and an appropriate solvent content is provided to the *TRUNCATE* procedure. In the absence of this information a solvent fraction of 50% is assumed, with the remaining volume filled with ‘average’ protein (as described in the *TRUNCATE* manual page).

## 5. Implementing automated data reduction   

Up to this point, the emphasis of this paper has been on the decision-making protocols for MX data reduction. To provide a useful tool for the user, these decisions must be embedded in a framework which expresses the overall workflow of the data analysis. In this section, the technical details of how *xia*2 works will be described.

### 5.1. Data management   

As an expert system can only make decisions and perform analysis based on the information available, careful management of all data is critical. Most of the data familiar to macromolecular crystallographers define static information, including coordinate and reflection files. Within *xia*2 some information is static, for example user input, but the majority of the information is dynamic in nature: the current state of hypotheses which are subject to change depending on the outcome of subsequent analysis. In the case of dynamic information it is important that their provenance is tracked, so that if a hypothesis is invalidated (*e.g.* the assignment of the Bravais lattice) all of the results derived from that hypothesis (*e.g.* the integrated data) are also invalidated. Within *xia*2 the approach to resolving this challenge is to maintain links to all of the sources of information and to ensure that information is freshly requested every time it is needed rather than stored.

The main information provided to the system is the raw diffraction data, with suitable metadata (*i.e.* image headers; detailed in Supplementary Material) to describe the experiment. Provided that the data are from a single sample or set of equivalent crystals this information will be sufficient to build a useful model of how the experiment was performed. Within *xia*2 the raw data are structured in terms of sweeps of diffraction data, which belong to wavelengths (which are merged to a single MTZ data set in the output), which in turn belong to crystals, which are finally contained within projects. Crystals are also the fundamental unit of data for scaling. All of these data structures (project, crystal, wavelength and sweep) map directly onto objects within *xia*2 and initially contain only static data: from this information it is possible to perform analysis using the procedures described earlier and to draw conclusions. In the situation where the data are not from a single sample or set of equivalent samples (for example, isomorphous derivatives recorded at a single wavelength) *xia*2 *cannot* know from the image headers alone how best to treat the data. In this situation it is necessary for the user to prepare a *xia*2 input file defining the logical structure of the data to be processed (see the *xia*2 manual for details.)

Within *xia*2 the analysis steps are performed by software modules (*Indexer*s, *Integrater*s and *Scaler*s), each of which have a well defined responsibility within the processing workflow. As described above, there will be situations where the analysis in one step will be dependent on the results of a previous step, and as such a reference to these results must be kept. This is greatly simplified if all implementations of a given function (*i.e.* indexing, integration and scaling) share a common description: in software terms they share a common interface. Within *xia*2 links to information are maintained *via* this common interface, greatly simplifying the bookkeeping components of the software.

### 5.2. Expert system interfaces   

The use of abstract interfaces for software modules is a common paradigm in modern software development, as other software using that module does not need to know about the internal details. In particular, the module may be replaced with another instance sharing the same interface with no loss of functionality. Within *xia*2 abstract interfaces are defined for the key analysis steps, with the intention that the modules which perform these key analysis steps (*e.g.* wrappers around *MOSFLM*, *LABELIT* or *XDS*) interact only through the abstract interfaces. As an example, both *MOSFLM* and *LABELIT* may be used to index a diffraction pattern based on a small number of images from a sweep, so both may present an *Indexer* interface. The details of how the indexing is performed and how the results are interpreted will be program-specific and are implemented in *MosflmIndexer* and *LabelitIndexer*, respectively. This approach has several advantages. Firstly, new modules may be added to the system without modification of the rest of the system. Secondly, code that is common to all implementations of a given interface may reside within the interface definition rather than being duplicated. An example of this is the handling of indexing solutions, where the management of the solutions and the handling of Bravais lattice solutions is in the *Indexer* interface code. Finally, this simplifies the two-way connections between modules, as they can share a common ‘language’. This is particularly important where, for example, a Bravais lattice choice is found to be incorrect late in the analysis: the behaviour of how to recover this situation is independent of the indexing software used.

**Figure 7:**
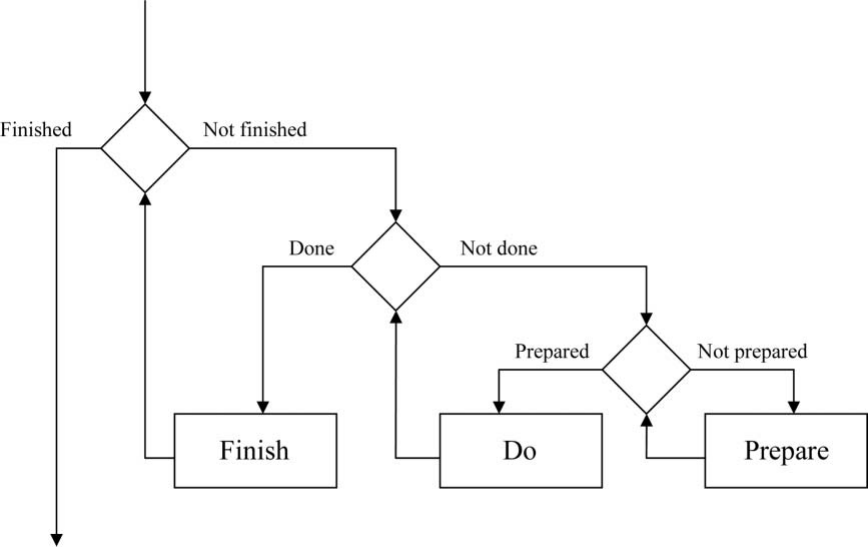
General flow of expert system interfaces, showing how the prepare, do and finish functions are used to ensure that all prior tasks are completed before a new step is initiated

Finally, it was decided that each analysis interface would be split into three phases, prepare, do and finish, arranged within loops (Fig. 7 ▶). The status of each phase is managed within the module and each phase will be performed until it is successfully completed or an error occurs. Once the finish phase is completed it will be assumed that all results derived are valid until proven otherwise, which will be verified when the results are requested. If subsequent analysis indicates that a result is incorrect this will be flagged and a new result calculated in response to the next request. Any change in the input may also invalidate the internal state, ensuring that the next time the results are requested they will be recalculated taking this change into consideration. While such a structure may appear to be complex, it has real benefits when linked to the data hierarchy described in §5.1.

### 5.3. Linking data structures and interfaces   

The benefit of having standard interfaces to the key analysis steps and clear links to the data hierarchy is that the source of any particular piece of information is well known. In *xia*2 links are made from objects in the data hierarchy to analysis modules which act on these objects, namely from sweeps to *Indexer*s and *Integrater*s and from crystals to *Scaler*s. This means that if a sweep is asked for the unit cell and Bravais lattice it may delegate this request to the *Indexer*. If no indexing has yet been performed or the solution has been invalidated then the sweep may be indexed automatically to provide the result. If a valid result is available this will be returned immediately.

This structure means that all analysis is performed when the results are needed and not before, ensuring that no unnecessary processing is performed. The loop structure of each module ensures that invalid results can be recalculated before the processing can proceed. In addition, the second outcome of this structure is that, since the dependency relationships between all pieces of information are well known, the processing necessary to deliver a result is performed implicitly. The result of this is that the ‘main program’ of *xia*2 is in effect a print statement, with the processing performed to provide the results to print.

## 6. Discussion   

The growth in the field of MX and the emphasis on answering biological questions has led to a strong push for the develop­ment of high-throughput techniques. One outcome of this has been the emergence of a new class of user of MX: the biologist using MX as a tool without themselves being an expert crystallographer. This has created a demand for more expert tools to help the user to collect and analyse their data and to solve and refine their structures. *xia*2 provides a platform for the reduction and analysis of raw crystallographic data and embeds within it expertise on the use of several well respected data-reduction packages. By encoding decisions as hypotheses to be tested as analysis proceeds, *xia*2 has the flexibility to use results from all steps in the analysis. The architecture also allows extension to include new software as and when it becomes available to adapt to the demands of crystallographers and to keep pace with new developments.

The decision-making protocols here reflect a systematic study of processing options for structural genomics data. Many of the conclusions of this reassuringly reflect ‘common sense’; for example, following the recommendations from the authors of *MOSFLM* on integration protocols with that program! Other outcomes suggest that small changes in established protocols can result in improvements to the reliability of the processing or to the accuracy of the results. As such, even with interactive processing with, for example, *iMosflm* these suggestions may be useful. While the fact that the study was performed with structural genomics data may suggest some bias to the effectiveness of *xia*2 with less than ideal data, experience (and citations) suggest that the results derived here have more widespread applicability.

## Supplementary Material

Supporting information file. DOI: 10.1107/S0907444913015308/ba5195sup1.pdf

## Figures and Tables

**Table 1:** Average *R*_merge_ values for processing of 38 sweeps of diffraction data with *MOSFLM* following five integration protocols, each normalized to the value resulting from the recommended protocol Clearly, performing cell refinement during integration is less reliable (in general) and gives poorer results. Otherwise, adjustments to the recommended protocol have little effect.

Integration protocol	*R*/*R*_recommended_	Success	Failure
(1) Recommended	1.00	38	0
(2) *P*1 + cell refinement	1.15	14	24
(3) *P*1, no cell refinement	1.00	38	0
(4) Recommended + resolution limit	1.00	38	0
(5) Refine cell with lattice constraints	1.23	17	21

**Table 2:** Average *R*_merge_ values for processing of 38 sweeps of diffraction data with *XDS* following five protocols, each normalized to the value corresponding to generate_XDS.INP Clearly, all protocols are reliable and offer only small differences in the accuracy of the measured intensities, although the recycling of the reflection-profile parameters gave a measurable improvement.

Integration protocol	*R*/*R*_recommended_	Success	Failure
(1) generate_XDS.INP	1.00	38	0
(2) + lattice constraints	1.00	38	0
(3) + recycle GXPARM	1.00	38	0
(4) + recycle profile parameters	0.98	38	0
(5) + recycle all corrections including detector distortions	1.02	38	0

**Table 3:** Eight different scaling models tested for the 12 JCSG data sets, corresponding to the normalized merging residuals shown in Fig. 6 ▶ The first run corresponds to a very simple scaling model, while the last includes all corrections and run 4 corresponds to the *CCP*4*i* default.

Scaling run	Partiality correct (*i.e.* ‘tails’)	Decay correction (*i.e.* ‘bfactor on’)	Absorption correction (*i.e.* ‘secondary 6’)
1	No	No	No
2	No	No	Yes
3	No	Yes	No
4	No	Yes	Yes
5	Yes	No	No
6	Yes	No	Yes
7	Yes	Yes	No
8	Yes	Yes	Yes

## Footnotes

1 Supplementary material has been deposited in the IUCr electronic archive (Reference: BA5195). Services for accessing this material are described at the back of the journal.
