# Supplementary material for: Decision making in xia2
Source: Acta Crystallogr D Biol Crystallogr. 2013 Jun 18;69(Pt 7):1260–73. doi: 10.1107/S0907444913015308 (PMC3689529; doi:10.1107/S0907444913015308)
Supplement: Supplementary file 1 [file d-69-01260-sup1.pdf]

Supplementary Material

Supplementary Table S1. Test data sets taken from JSCG

Sample ID	PDB ID	Multi-pass	Multi-wavelength	Resolution
9172	1VK8	X	X	1.8
10230	1VKW	X	X	2.0
12287	2ISB	-	X	1.7
12847	1VR9	X	-	1.7
13140	1VR9	-	X	2.0
45453	2PBL	-	X	1.8
7612	1VKE	X	-	1.6
7644	2ETH	X	-	2.5
7722	1VK9	-	-	2.4
7797	1VKK	X	-	1.4
8582	1VP4	-	X	3.2
9168	1VK9	-	X	2.7
9781	2A0N	X	-	2.1
9969	1VK2	X	X	1.9
10175	1VMA	X	-	1.6
10203	1VKF	X	X	1.9
10301	1VME	X	X	1.8
10350	1VKF	X	-	1.7
10401	2A0N	X	-	1.6
11258	1VPY	-	-	3.1
11580	1VP4	-	-	2.2
11859	1VPY	-	X	2.5
12316	2PWN	-	X	2.0
12972	1Z85	X	X	2.1
13089	1VR7	X	X	1.3
13120	1Z82	-	X	2.0
13185	1VRM	-	X	1.6
13193	1VR5	X	X	1.7
17403	2GB4	X	-	1.25
27032	2P4G	-	X	2.3
29928	2HXV	-	X	1.8
29929	2HXV	X	-	1.8
33102	2P4G	-	-	2.3
33758	2PFX	-	X	1.7
39191	2Q02	-	X	2.4
39621	2PFW	-	X	1.9
44156	2PPV	X	X	2.0
44283	2PNK	-	X	2.0
47717	2PYQ	X	X	1.5
52009	2QYV	-	X	2.1
56883	2R6V	-	X	1.3
58046	2RH0	-	X	2.0

Sample ID	Point group symmetry	a	b	c	alpha	beta	gamma
9172	P1	47.6	47.7	49.6	73.8	62.9	73.6
10230	P321	45.3	45.3	193.1	90.0	90.0	120.0
12287	P422	51.7	51.7	157.9	90.0	90.0	90.0
12847	C2	228.6	52.7	44.1	90.0	100.5	90.0
13140	I222	43.6	52.2	222.6	90.0	90.0	90.0
45453	P2	77.2	74.4	95.7	90.0	113.4	90.0
7612	P2	67.9	68.0	90.1	90.0	97.5	90.0
7644	C2	159.4	117.4	85.1	90.0	105.7	90.0
7722	P222	58.4	73.5	93.1	90.0	90.0	90.0
7797	P1	29.0	34.6	38.4	70.0	87.5	70.5
8582	P1	166.3	166.8	68.4	94.0	93.0	118.3
9168	P622	132.9	132.9	66.4	90.0	90.0	120.0
9781	C2	96.7	96.7	154.2	90.0	90.0	120.0
9969	P422	61.2	61.2	127.7	90.0	90.0	90.0
10175	P2	78.5	48.2	90.9	90.0	107.9	90.0
10203	P622	85.3	85.3	92.6	90.0	90.0	120.0
10301	P2	55.2	95.8	90.1	90.0	95.4	90.0
10350	C222	84.6	138.9	160.7	90.0	90.0	90.0
10401	P622	96.6	96.6	155.8	90.0	90.0	120.0
11258	P222	91.8	92.4	119.3	90.0	90.0	90.0
11580	P222	65.6	116.6	78.1	90.0	90.0	90.0
11859	P6	114.2	114.2	52.4	90.0	90.0	120.0
12316	P321	77.5	77.5	56.6	90.0	90.0	120.0
12972	P222	65.5	82.3	106.0	90.0	90.0	90.0
13089	H3	105.4	105.4	69.5	90.0	90.0	120.0
13120	P2	65.4	67.6	75.8	90.0	113.5	90.0
13185	P222	57.7	77.0	86.6	90.0	90.0	90.0
13193	P222	140.0	96.6	115.8	90.0	90.0	90.0
17403	C222	63.0	70.6	72.6	90.0	115.7	90.0
27032	P6	115.7	115.7	56.9	90.0	90.0	120.0
29928	I422	104.5	104.5	146.6	90.0	90.0	90.0
29929	I422	104.2	104.2	145.9	90.0	90.0	90.0
33102	P6	113.5	113.5	55.9	90.0	90.0	120.0
33758	P6	85.2	85.2	105.6	90.0	90.0	120.0
39191	P222	94.7	95.4	136.9	90.0	90.0	90.0
39621	P422	73.1	73.1	58.4	90.0	90.0	90.0
44156	C222	136.7	136.8	56.0	90.0	90.0	90.0
44283	C2	273.8	158.6	181.3	90.0	116.0	90.0
47717	P2	33.0	99.2	58.7	90.0	90.4	90.0
52009	I222	173.9	84.3	123.2	90.0	90.0	90.0
56883	P622	46.2	46.2	267.6	90.0	90.0	120.0
58046	C2	44.6	64.5	64.4	74.5	81.1	81.

Selection of images for autoindexing and cell refinement with MOSFLM, LABELIT and XDS

MOSFLM and LABELIT

To optimise any process a scoring scheme is needed. Here the objective is to determine the selection of images which generally give the most accurate indexing solution. As the minimum root mean square (R.M.S.) deviation between observed and predicted spot centres is a target of refinement and the ideal absolute values of the unit cell constants are poorly defined (at least at this stage) these both represent poor metrics. The metric penalty however, defined as the deviation from the constraints for each Bravais lattice (Grosse-Kunstleve, Sauter & Adams, 2004), is appropriate as this is a test for internal consistency and is also relevant for automatic strategy systems. With the possibility of characterisation prior to data collection in mind, the use of few images may also be desirable.

Data were taken from 86 sweeps from the JCSG archive, where the following four criteria were met: the lattice was not pseudosymmetric, nor triclinic, autoindexing with a single image gave a reasonable result and at least 90° of data were available. The resulting sweeps had resolution limits in the range 3.5Å to 1.2Å. The number of images to use for indexing was considered first. Figure S1 shows the mean normalized metric penalty, calculated from all of the metric penalties for a given sweep (i.e. for indexing from 1 - 15 frames) scaled over the range 0 - 1, averaged across all sweeps for each number of images. The calculations were performed for images spread across the range 0 - 90°, and smaller values indicate a more accurate solution. Clearly the use of two images (≈ 0.24) gives a substantially more accurate result than the use of a single frame (≈ 0.92), confirming the advice from the MOSFLM and LABELIT authors. However a further improvement may be observed from using three images (to ≈ 0.14), after which no further improvement is clear. Following a similar procedure it was found (Figure S2) that a spacing of more than ≈ 20 - 30° generally gave the most accurate solution with a minimum ≈ 45°. Extending this analysis to sweeps of up to 180°, allowing spacings to 90°, confirmed this result (Figure S3) .

These results may be considered as follows. The one-dimensional FFT indexing procedure employed in MOSFLM and LABELIT computes the Fourier transform of the projection of the observed peaks gathered from the selected images in ≈ 7000 reciprocal space directions. Those directions which show the strongest signal are then considered as possible basis vectors. Using three images spaced by 45° ensures that every reciprocal space direction is likely to be well sampled. This will make it more likely that a fundamental basis vector, rather than some linear combination of basis vectors, will be found giving a more accurate result.

XDS

By anology with LABELIT, data were indexed with a triclinic basis from all images and from one to ten 5° wedges, with the resulting triclinic cell used to compute the metric penalty for the correct lattice using tools from CCTBX. As may be seen from Figure S4, use of a single 5° wedge gave the least accurate results followed by the use of all images. The use of two and three wedges showed improved accuracy, with no improvement observed subsequently. Following from this, various wedge sizes were tested and no substantial trends, beyond using at least two frames, were found though a slight benefit was observed for using ≈ 5° wedges (Figure S5) Finally the ideal spacing was found to be in excess of ≈ 20 - 30° (Figure S6) with a 45° spacing used if possible.

MOSFLM cell refinement

Within xia2 the cell refinement with MOSFLM is performed between the initial indexing and integration. It performs two functions: to refine the parameters prior to integration and to test the validity of the autoindexing solution. Both of these functions are best achieved if the resulting unit cell parameters are accurate, which may be assessed as above by performing the refinement with a triclinic unit cell and computing the metric penalty. Following similar protocols to the XDS indexing, Figures S7, S8 and S9 show the mean normalized metric penalty as a function of the number of wedges, the number of frames in each wedge and the wedge spacing. Clearly the use of two or more wedges is critical, and the use of 3 – 4 images per wedge and rotation between wedges in excess of 15° should work well. These are very similar conclusions to the XDS indexing analysis.

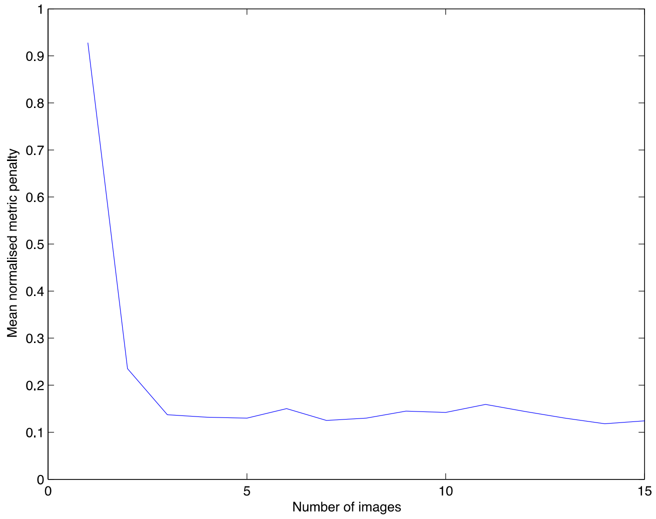

Supplementary Figure S1. Mean normalized metric penalty as a function of the number of images used for characterisation with LABELIT, where smaller values indicate more accurate solutions.

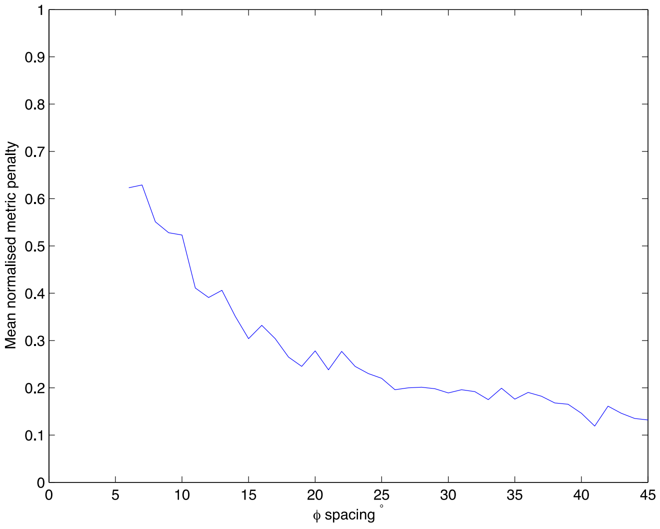

Supplementary Figure S2. Normalized metric penalty for indexing from three images with LABELIT as a function of image spacing, up to a maximum of 45°.

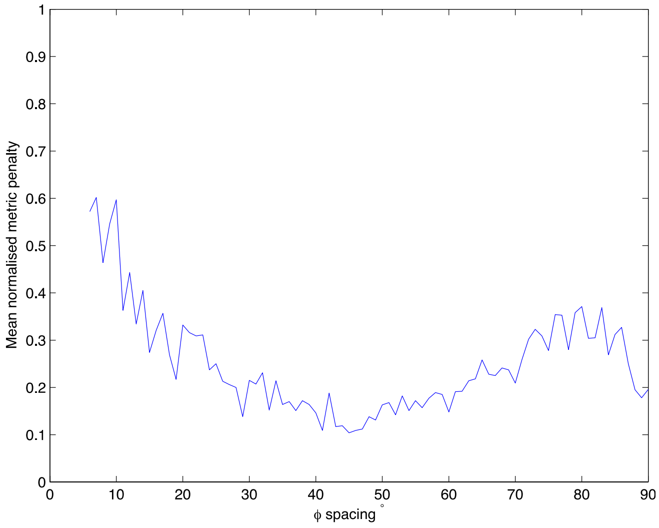

Supplementary Figure S3. Normalized metric penalty for indexing from three images with LABELIT as a function of image spacing up to a maximum of 90° (with fewer examples than Figure S2).

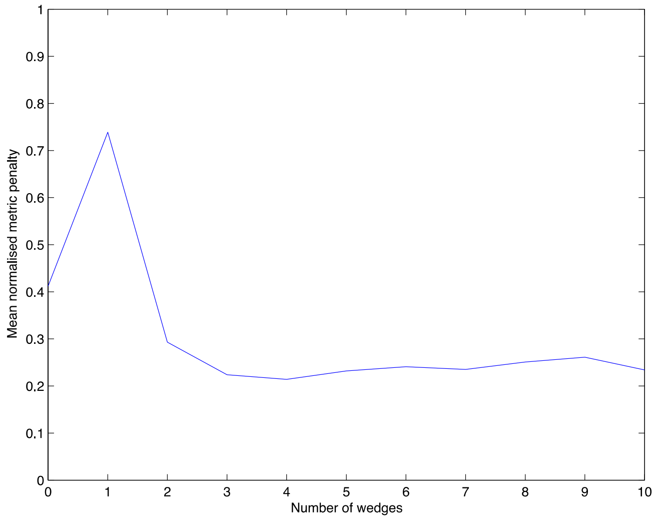

Supplementary Figure S4. Mean normalized metric penalty for the correct autoindexing solution from XDS, as a function of the number of 5° wedges used. The data point 0 corresponds to the use of all images.

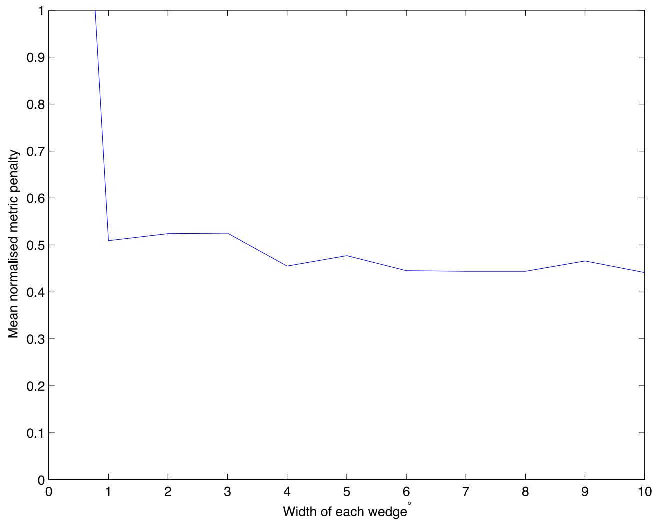

Supplementary Figure S5. Mean normalized metric penalty as a function of wedge width, showing the benefit of using at least two images.

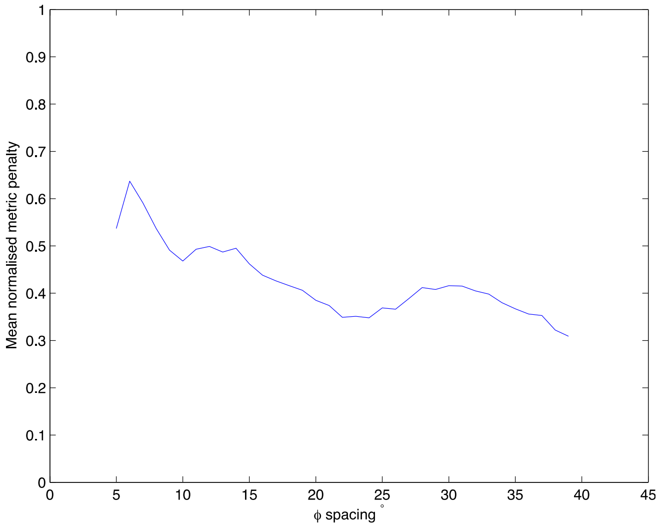

Supplementary Figure S6. Mean normalized metric penalty as a function of wedge spacing, showing generally more accurate results for spacings wider than around 15°.

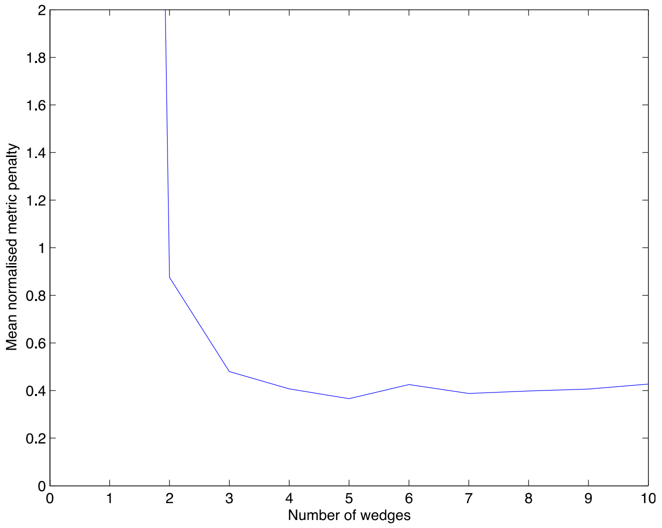

Supplementary Figure S7. Mean normalized metric penalty calculated from triclinic cell parameters resulting from cell refinement with MOSFLM, as a function of number of wedges: clearly having two or more wedges is critical.

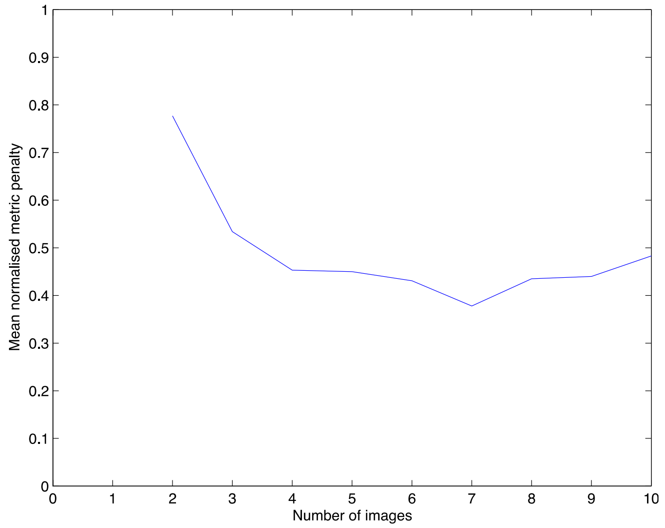

Supplementary Figure S8. Mean normalized metric penalty for three wedges in MOSFLM cell refinement, showing an advantage to using three or more frames in each wedge.

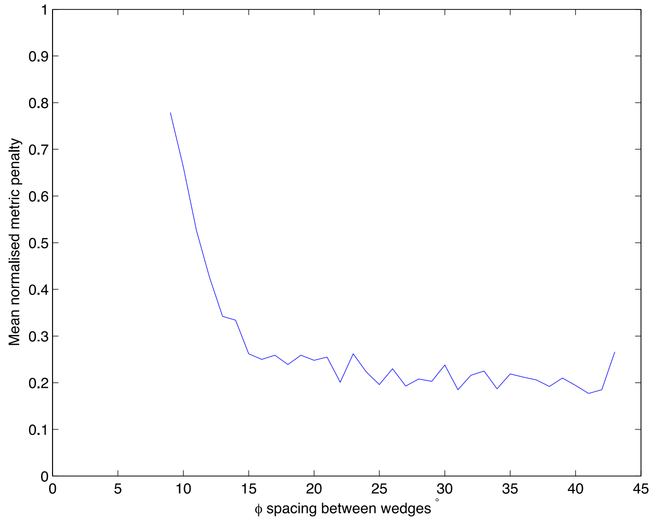

Supplementary Figure S9. Mean normalized metric penalty for three wedges of three images in MOSFLM cell refinement, as a function of angular spacing between the wedges. Clearly (like XDS autoindexing) using a spacing of 15° or more is beneficial.

Required contents of image headers

For the automated processing to be successful the following information is assumed to be present in image headers, and is assumed to be essentially correct: the timestamp, pixel size, exposure time, detector dimensions (i.e. number of pixels in each direction), the wavelength, detector distance, beam centre, the oscillation angle and the oscillation range. This data is typically present and correctly supported by xia2 for instruments manufactured by: Dectris, Rigaku, ADSC and Rayonix.
